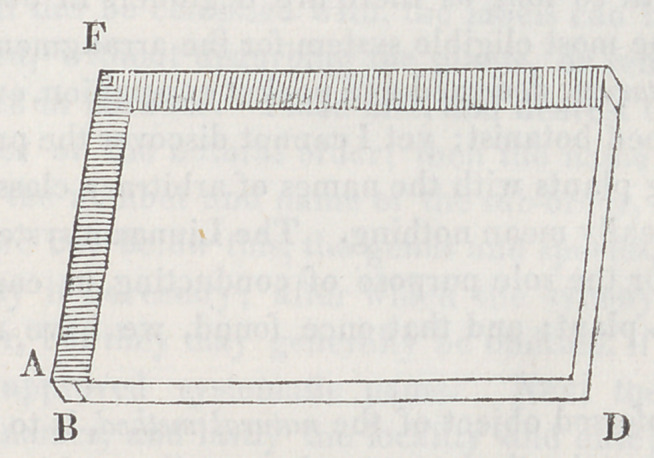# Botanical Specimens

**Published:** 1834

**Authors:** John L. Riddell


### Art. III.—Botanical Specimens.

*Particular Directions for Collecting and Preserving Specimens of Plants, extracted from an unpublished treatise on Practical Botany.* By John L. Riddell, A. M.*

Those who are about to make a botanical excursion, are generally recommended to provide themselves with a tin box, near 18 inches long and 6 wide, which some have termed a *vasculum*. Such a box, being impervious to moisture, and an excellent reflector of the light and heat of the sun, is admirably calculated to preserve flowers and leaves in a perfect state of freshness, for a very considerable time. If a few delicate specimens only, occupy the box, they will be sure in a short time to languish or become crisped, unless we take the precaution to accompany them with a liberal quantity of very damp moss, or something equivalent to it. For the exhalation of moisture from the leaves and flowers, continues so long as there is moderately dry air in the box to receive it; and as

* Every day diminishes the number of individual specimens, and, perhaps, every year annihilates several species of the plants indigenous to the Valley of the Mississippi; it is, therefore, a matter of the deepest interest, to direct the attention of our people to the study of Botany. This we have more than once attempted to do, by reviews, notices, and exhortations, in this Journal; but with no very encouraging success. As a means of rousing up our students of medicine, and young physicians, many of whom (like some of their seniors) have occasionally a little leisure on hand, we insert the following extract from the work which Mr. Riddell proposes in due time to lay before the public. We hope he will append to his practical directions, a catalogue of such of the plants of the State of Ohio, as may have fallen under his observation. He is, we feel assured, a sound practical Botanist, who may, perhaps, do for Ohio, sooner or later, what Professor Short is zealously laboring to effect for Kentucky. By the way, why does not the Professor bring out the Flora, for which he must have on hand such ample materials? Its publication would do more to *promote* the study of Botany in the basin of the greatest of rivers, than a thousand of our paragraphs. Editor.

the plants can no longer suck up fluids from the earth, they become exhausted by the immediate loss they sustain.

Although some esteem the vasculum very highly, and employ it exclusively in their herborizations, for myself I disagree with them in opinion, and give my decided preference to the *port-folio.** I shall hereafter assign some of the reasons which have influenced my choice.

The following articles will be found subservient to the prosecution of our object.

1. A common mason's *trowel.*† This, or an implement somewhat similar to it, will be found almost indispensable, for procuring herbaceous plants by the roots; which should never be neglected when it can conveniently be done. When one preserves small specimens merely to assist the memory in recognizing and distinguishing species, the roots may generally be dispensed with. But an herbarium designed for scientific reference, would indeed be *radically* deficient, if it did not comprise the roots of most herbaceous plants, preserved, either entire, or in thin sections. As to the roots of trees and shrubs, they are so little diversified in character, and withal so unmanageable on account of size, texture and situation, that we must generally remain contented to leave them undisturbed. The trowel is equally useful and more convenient, if rather smaller than the usual size. As a substitute for this instrument, one may use a butcher's knife, with a broad blade and dull edge.

2. A sharp *Jack-knife;* for trimming specimens, cutting them of the proper length for the port-folio; making sections of thick roots, coarse fruits, capsules and the like, and for lopping off the branches of trees.

3. A small sharp *pen-knife*, or *surgeon's lancet;* for making dissections of flowers.

*If a person were riding through a country, in such haste that he had not time to place his specimens properly in a port-folio, the vasculum would undoubtedly be preferable.

†"Also a *bill-hook*, fitted to screw into a cane."
*Practical Naturalist.*

4. A *portable volume*, containing brief descriptions of the plants which will probably be met with in the excursion. The more local a manual of descriptive botany is, the more convenient will it be found in the particular region to which it has been adapted. In other words, the student will find it more difficult to determine the names of plants with which he is unacquainted, in proportion to the extended number of species among which he has to decide. Dr. L. C. Beck, in his accurate work on botany lately published, gives descriptions of the plants indigenous to the Northern and Middle States. Professor Eaton's Manual, 6th Edition, includes all the known indigenous species, north of the Gulf of Mexico, and many cultivated exotics. This book, from its being so portable and comprehensive, will doubtless be found more convenient to students of botany in the Western States, than any other volume extant. As yet we have no separate Flora of western plants.

5. *Magnifying glasses;* for aiding in the analysis of plants, by rendering the organs in minute flowers more obvious and easy to be examined. An instrument possessing a magnifying power of 10 or 12 diameters, will be amply sufficient in nearly all cases. Indeed, in most instances the eye will not require the help of glasses. The student would do well to purchase either the common magnifier, which consists of three lenses put up in horn; or the very convex, single lens, mounted in brass, and accompanied with little forceps.

6. *Pencil* and *paper* for labelling.

7. When on any considerable excursion, one should be provided with a *note book*, pen and ink.

8. A *bag* of pliable *oil cloth*. It should be nearly two feet square. To make it, would of course require eight square feet, or nearly a square yard of cloth. Brown Holland, or very strong silk, is a good material to make one of. After having been drawn smooth and secured, in a situation where the sun's rays can have access to it, it should receive two or three successive coats of drying linseed oil. After becoming dry, it may be sewed into a bag, the opening or mouth of which, like its width should be two feet. Perhaps the cloth should be black, in preference to a lighter color. The particular hue is a matter of very little importance I imagine, for though dark colors will exclude more of the sun's light, they are much better adapted to receive radiant heat from the same source, than lighter ones.

The oil cloth bag if well made, is exceedingly useful. It receives the specimens when first collected, where they will lie perfectly secure for a considerable time, as in the vasculum. It possesses some advantages over the latter apparatus; viz. Its capacity may be easily graduated to its contents. When not in use it may be folded up and stowed away in a pocket, or in the port-folio; while the dimensions of the tin box will admit of no such abridgment. On the other hand, though the oil cloth effectually prevents evaporation, it does not entirely exclude the influence of the sun. But this will seldom become a source of evil, for when the collector has procured a sufficient number of specimens, he will seat himself in a convenient place and transfer them to the port-folio.

While travelling, one may keep plants fresh in an oil cloth bag, from 10 to 30 hours; in a tin box for a number of days. Should a deep river oppose the adventurer's progress, he has only to tie his equipments and clothing snugly in the impervious bag, attaching a few feet of string to it, the end of which he will hold in his mouth, and like a bold and experienced swimmer, commit himself to the embrace of the waters. Should showers of rain meet him at a distance from human habitation, the oil cloth serves to protect his plants, and books, and other articles from injury.

9. A *port-folio*. This should be a large morocco, or leather bound book, containing a quantity of soft bibulous paper. The most eligible dimenions are 15 or 16 inches in length, by 10 or 11 in breadth, measuring when the book is closed. Soft wrapping paper, or common printing paper, is a very good material for the leaves, which should be near 150 in number; thus making a book of about 300 pages. The covers should contain moderately stiff binder's boards, and the whole should be put together after the manner of a full bound book, with certain modifications about to be mentioned. The back should be near twice the thickness of the front part, and this perhaps can be most neatly accomplished by first making a book of 600 pages, then removing one half of the leaves with a knife. To a flap of leather, extending from one of the covers, after the manner of a pocket-book, a buckle is permanently attached, and from this a strap descends and terminates in a hook, which is received into a ring or eye, thus allowing the port-folio to be opened or closed with the greatest convenience. Buckles should also be attached, for the reception of a strap designed to pass over the shoulder.

In this manner the port-folio can be carried with the greatest ease and convenience, while the plants will be completely protected from that mischievous compression, which they would be liable to suffer if carried under the arm.

The port-folio is not designed for drying plants; only for safely keeping them while on an excursion. Indeed, if plants are to be retained some three or four days in the port-folio, it is nearly always an essential object to prevent their drying.

If small specimens only are required, the port-folio may be of a size correspondingly small. It is hardly necessary to add that a large printed volume, or old account book may be used as a substitute.

*Directions for placing specimens in the port-folio.* I shall be considerably minute and explicit in my advices on this point, for on the skilful accomplishment of this mechanical part, depend in a great measure the beauty and usefulness of an herbarium. Having selected the requisite number of the most perfect specimens to be found, and having washed or beaten the earth from the roots, the botanist is to seat himself as comfortably as circumstances will permit, and proceed to cut down the larger plants to proper dimensions, remove tuberosities from the roots and stem, divide compound flowers,

&c. in the manner hereafter to be pointed out. The specimens are to occupy a place conveniently within reach on his right. The port-folio is to be opened, and that lid of the cover to which the flap of leather is attached, must be placed on the right knee, the other of course on the left. When thus arranged, the leaves of the port-folio rest on the left lid. A specimen is to be taken in the right hand, and the first leaf of paper in the left; when a flower or any other part of the specimen is placed in a proper position by the right hand, that position is to be secured by bringing the sheet of paper over it with the left hand. The left arm or wrist will now maintain this portion of the paper in place, while the hands are at liberty to adjust other parts of the specimen in the same manner. The second leaf of paper is to overlay the next specimen, and so on until the specimens are all exhausted. Sometimes a pen-knife or sharp stick will be found very convenient, in arranging minutely subdivided leaves, or the parts of complicated flowers. After a little practice, one can put up specimens in a manner perfectly neat, and with a degree of despatch that is almost incredible.

It is usually advisable to place leaves, so that their upper surfaces will all look the same way. If the leaves are small, and the two surfaces very similar in appearance, as in Gerardia tenuifolia, Lythrum hyssopifolium, this particular will need little attention.

If roots are too thick to find a place in an herbal, they may be pared down, or thin sections of them may be saved. It is better to preserve such roots by washing them carefully in the first place, and allowing them to dry in the open air, attaching a name or number to them by means of a needle, strong thread or twine, and a piece of card.

Thick succulent fruits, as the pawpaw, and persimon, are best preserved in spirits of wine; but is is most always advisable to place transverse and longitudinal sections of them in the port-folio. It is certainly very well to accompany the twigs, leaves, and flowers of forest trees and shrubs, with neat sections of the wood and bark. The bark of the trunk may be obtained with a knife, and the section should be a vertical one, and towards the centre of the tree. The wood is most conveniently procured from a branch of moderate size. Thin sections too, of such capsules as are borne by the Poppy, Nigella, and the like should be preserved.

Dissections of flowers can be rapidly and neatly made, with a small sharp knife or lancet; and they commonly present a beautiful appearance when dry. To prevent the stamens, styles, sections of immature capsules, and other minute parts, from being lost, they should be immediately laid on a piece of fine gummed paper, and placed in the port-folio. The pieces of paper ought generally to be one inch in width and two inches long. The dissections commonly moisten the gum by the juices they contain, and after the whole has been subjected to the drying process, remain adherent. All the organs should be thus displayed upon which generic distinctions depend, as the calyx, corol, stamens, pistil and germ; and in compound flowers, like those of Prenanthes and Helianthus, the aigrette chaff, receptacle and seed. Indeed, thick compound flowers, as those common to the genera Silphium, Helianthus, Rudbeckia, Dahlia, cannot be well preserved without the assistance of the knife. It is commonly necessary to sever one half of the flower by a vertical section, and then to remove a number of the disk florets with the fingers. When thus divided, the flowers are easily adjusted in the port-folio, and when dried are very beautiful and well adapted for reference, exhibiting as they do the generic characters to great advantage. Either side of the mutilated flower may correspond to the upper surface of the leaves, according to the fancy of the collector.

Before roots are placed in the port-folio they should be washed in water, and most of the superficial moisture removed by the application of spare bibulous paper. Marine plants, and aquatic vegetables inhabiting stagnant pools, likewise require careful washing in clean fresh water, and a partial drying as before, ere they can be admitted into the port-folio. At this time the parts of these plants are easily arranged, and few specimens will exceed in beauty the Algæ, Ceratophylleæ, and Lentibulariæ when properly adjusted.

In regard to the *size* of specimens, all small plants are to be taken entire. Larger ones must from necessity be abridged and divided until the port-folio will conveniently receive them. Take for example a tall plant as the Solidago rigida. One specimen should embrace the root and radical leaves, another the corymb of flowers and the cauline leaves. As to the *number* of specimens, six or eight extras should generally be taken for the purpose of exchanging; and if we meet with plants that are rare, or peculiar to certain localities, a still greater number should be secured.

Having selected and put up the requisite number of specimens for preservation, we are next to set about determining the name from a pocket manual or Flora. A mere glance at the flower will generally enable one to determine the Linnæan class and order, while the neat dissections mentioned above, will materially aid in referring the plant to its genus. In hunting for the specific description, it is seldom or never necessary to read all that is said under each species—but we should avail ourselves of some prominent character which the plant possesses, and with it test each description. Suppose the leaves of our plant to be heart-shaped. Now this circumstance will enable us almost at once to exclude it from all those species that do not possess cordate leaves; and thus our labor will be very much abridged. After we have had some little experience, the names of most plants may be thus determined in a few minutes. The name, locality and date, are to be written on a slip of paper, which is to be placed in the port-folio with the specimens. If any doubt remain as to the correctness of our analytical result, a mark of interrogation (?) is to be affixed to the generic or specific name, as the case may be.

If we have reason to suppose that the plant is unknown to botanists, it is incumbent on us to insert a pretty full description of it in the note book; referring to the specimens by a number. For the sake of system, it is perhaps best to describe, 1st. The root. 2. Stem and branches. 3. Buds and foliation. 4. Leaves. 5. Appendages. 6. Inflorescence. 7. Calyx. 8. Corol. 9. Stamens. 10. Pistil. 11. Pericarp. 12. Seed. 13. Receptacle.* Then add an account of the size and general appearance of the plant, the points of resemblance it has to well known species, the situation and nature of the soil in which it grows, time of flowering; and as such particulars are generally omitted in the systematic description of the parts above referred to, do not here omit to record the color of the flower, the stem and the leaves; and the duration of the root if it can be determined, whether annual, biennial, or perennial.

Plants should not be subjected to pressure while in the port-folio, for their vitality is thereby the sooner destroyed, and as a consequence they will be likely to undergo some modification in color, detrimental to their appearance, before they are transferred to the press. The plan of carrying the port-folio suspended from the shoulder, is calculated to avoid this unpleasant occurrence.

When a botanical excursion occupies some days, the leaves of the port-folio will probably become filled with plants before we have collected one fifth of the number we mean to procure. In this case we may transfer the specimens to the part of the book first occupied, laying five, six, or more, between every pair of contiguous leaves; and all this engrosses but little time, for the leaves and flowers have now become so stiff, that they retain the relative position in which they were first placed: When thus packed over, they are to be secured by the oil cloth flaps.†

No pains should ever be taken to dry the port-folio, for it is better that its leaves should be moderately damp. When recent plants are to be transported in it a considerable distance, or retained in it a number of days, it is advisable to envelope it closely in the oil cloth bag, in order to prevent the exhalation of moisture.

*** Some prefer beginning with the calyx.**

**† The description of which is omitted in these extracts.**

*Times for procuring plants.* As a general rule, plants should be procured when fully in blossom; not only because they are most interesting and beautiful at this time, but for the more substantial reason, that they display those characters by which they are aggrouped into classes, orders and genera. It is better to get specimens of most forest trees at two periods; procuring the flower as it appears early in the season, and the leaves a few days before they attain their full size. No amateur in our science, however, will fail to collect leaves still later in the season, when they are richly variegated with autumnal tints.

The willows are quite numerous in the State of Ohio, and considerable difficulty will be encountered in obtaining complete specimens of them. The catkins appear before the leaves are developed, and when the leaves are mature, the staminate catkins are gone. I would recommend collectors to adopt the following method: Procure the flowers when they appear, and number the tree which yields them, with a knife or other sharp pointed instrument; accompany the specimens with the same number on a slip of paper. When the leaves are mature, collect and number them also. The leaves of the wild poplars may be collected in the same manner.

Compound flowers should be preserved in different stages of maturity, and the required specimens can often be taken from the same plant at the same time. The form of the seed, the nature of the aigrette, the condition of the receptacle, whether pitted, chaffy or smooth, can be determined most satisfactorily in mature specimens, from which the showy ray florets have fallen off.

The seeds and glumes of grasses are likewise required to be considerably advanced. All umbelliferous plants, as the dill, angelica and parsnip, should be gathered for preservation when the seed approaches maturity. It will certainly be well to preserve the recently expanded flowers if conveniently obtained, and they may frequently be found on the same stalk with the full grown umbel; but the mature seed is altogether most important, since the distinctive characters of umbelliferous genera, are mostly drawn from the furrows and ridges which variegate its surface.

Ferns are to be collected whenever the *sori* or fruit dots on the back of the leaves* are fairly developed. They are generally met with in this condition during summer and autumn, though a few hardy species as Aspidium marginale, A. acrostichoides, Pteris atropurpurea, are met with in the winter. Mosses are to be put up when their *thecæ* or little elevated urns have attained maturity. They will be found in some situations at all times of the year, but the greatest number will appear during damp weather late in autumn. The sprigs should be separated considerably, before being placed in the port-folio. Lichens are found most plentifully in swamps and in shady ravines, on trees and on rocks; and damp, cool weather is peculiarly favorable to their growth. Hence after a cold rain-storm late in autumn, they literally clothe the trunks and branches of trees, and also spring up luxuriantly from the earth. Whenever they bear a *thallus* or receptacle, resembling in form a button, a shield, a knob, or a cup, they may be admitted into the port-folio.

It often happens that the radical leaves (those springing from the root) of phenogamous plants possess a form and appearance different from those growing on the stem. The former, though sometimes obscure from their situation, should by no means be overlooked. If the plant belong to the Linnæan class Diœcia, as do the hop and the honey locust, specimens from both the staminate and pistillate individuals must be procured. Beginners, who really set about collecting plants in earnest, should omit to take nothing they find in season. They should not neglect plants because they are common, "because they are mere *weeds;*" nor refuse a rare specimen because it is a poor one. It should be an invariable practice to secure the first sample met with. It may afterwards be in one's power to substitute it by a better specimen if necessary. As a general rule, young botanists should take

* Some Ferns produce a fruit bearing spike or raceme.

specimens of those vegetables only, which are met with in flower or fruit. He who has already an extensive collection, will lay hands on every thing he sees that is new to him, in what condition soever he may find it.

It is strictly enjoined upon us by some, to abstain from collecting plants while they are moistened with dew or rain; an injunction which I would recommend no one to regard very closely. For if the water be shaken from them, or wiped from them with a fold of bibulous paper, they admit of being as well preserved as though collected under the most favorable circumstances.

*Directions for drying plants.* The next thing to be attended to is the drying process. If a loose bundle of herbs be hung up in an airy chamber, in a few days they will generally become quite dry. If the light be mostly excluded by closing the shutters, the leaves, and oftentimes the flowers, will retain their natural colors. The leaves, however, are sure to become rugose and contorted, and the petals crisped and deformed. To obviate these displacements, the fresh plant may be placed in a jar, and the vessel carefully filled with fine dry sand. The specimen imparts its humidity to the sand, while the sand faithfully retains its various parts in place. After this arrangement has been allowed to continue a few hours, the desiccation may be materially hastened, and with perfect safety to the specimen, by exposing the jar in some manner to a heat not exceeding 120 degrees Fahrenheit. Plants dried in this manner, cannot be admitted into the leaves of an herbal. They must be kept in a situation quite dark; or in time they will become blanched by the influence of light.

Specimens placed at intervals of 50 or 80 pages between the leaves of an old printed quarto, and subjected to moderate pressure, will become very well dried in the course of two or three weeks; and in the meantime they require no attention. Since the leaves of paper are invariably wrinkled or rendered uneven, no valuable book should ever be used for this purpose.

"*Directions for making an Herbarium.* 1. Provide yourself with about 100 old newspapers, or other coarse paper about equal to that quantity and texture. Let these papers be very thoroughly dried. This will be a sufficient stock for the season.

2. Procure two smooth inch boards of the size of half of a paper; also a weight of lead, stone or other substance, of 20 pounds.

3. Gather three or four specimens of each plant as it comes in flower. Place these between the folds of the papers, as nearly in their natural state as possible. If the plant curved, let it curve in the papers, if the flower drooped in the field or woods, let it droop in the papers, &c. Lay the papers between the boards with the weight upon them. If 20 or 30 filled papers lie upon each other, it is all the same.

4. Two or three times each week lay your papers, containing plants, separately in the sun, with small stones on the corners, for three or four hours. When taken in, put the plants in press again. This exposure to the sun is not necessary, however, with single specimens of small plants, or if several leaves of paper be allowed to each specimen."—*Eaton's Manual*, 5*th Edition.*

I shall now proceed to describe the fixtures and manipulations, which in my opinion are decidedly preferable, as being most convenient and successful in practice.

And first the *press*. Dr. Short recommends that the compressing power be applied by means of a screw, or a simple lever; and though it is a matter of no great consequence, so the pressure be justly applied, yet I prefer having recourse to the wheel-and-axle principle. With an apparatus of the latter sort, any amount of pressure can be conveniently commanded, while it possesses over the screw press the following advantages.

1. A greater simplicity of structure.

2. It occupies less space, and costs less money, though the expense of either is trifling.

3. It can be put in operation or dismounted with much less time and trouble, and this circumstance alone is of material importance.

4. When the dryers and plants are approximated by compression, the severity of the pressure is relieved in the screw-press, while in this it can be made to continue unabated.

The construction of the *axle-press* can be best understood by reference to the figures.

A, the bottom piece. B, the top piece. C, the axle. The bottom and top pieces may be 20 inches long, by 14 wide; 18 by 12 may do. They should be made from a seasoned board near an inch in thickness, possessing the requisite strength, and not liable to warp. The end pieces, I, H, J, are intended for maintaining the board a few inches from the floor, for the convenience of attaching or removing loops of rope. D, E, are the projecting ends of a strip of strong timber attached to the board beneath, over which the loops of rope pass. The ends of the longitudinal strip G F, are often useful in assisting to confine the lever soon to be mentioned. Transversely across the top piece and near the middle, two thin strips of board are to be strongly nailed, leaving an intervening channel or groove an inch in width. The axle is to lie in this groove, and if that be of iron, or any stronger material than wood, the transverse strips may be approximated still nearer with advantage. The smaller part of the axle should be a trifle less in diameter than the width of the groove above mentioned, and it should exceed in length the width of the press, by two or three inches. M and N are enlargements two inches in diameter, perforated in the same direction by holes for the reception of ropes. O is an enlargement having a diameter of four or five inches, and traversed by inch holes for receiving the lever. The ropes R and P pass through the holes at M and N, and are secured by knots. They are of equal length and terminate in loops. The mode of using this press must be obvious from the preceding account of it. I will only say, that the materials to be subjected to pressure are placed between the boards; the loops R and P, are attached to E, D; the axle is brought to its place, at the same time rolling the excess of rope upon it; a stout lever four or five feet long is to be put in place, and from its end a weight suspended, or a small cord extended and secured to one of the projecting pieces for that purpose.

Three or four *thin boards* of wood or paper, of a size corresponding to the press, will be found almost indispensable, whenever we have on hand a large number of undried plants. They not only subdivide the contents of the press into smaller parcels, and thus equalize the pressure, but serve to separate specimens that are nearly dry, from those that are yet replete with moisture.

Any kind of soft, unsized paper may be used for drying plants, as tea paper, common wrapping paper, printing paper and old newspapers. Near three reams will be required, and this quantity will amply suffice, unless one wishes to collect very liberally. Half a ream of this must be tea paper of a fine quality and large size, or something equivalent to it, while the remaining two reams and a half, may be coarse wrapping paper of a similar size. The more body this has, the more bibulous it is, and the greater freedom it possesses from little inequalities, the better will it be adapted for the purpose. *Dryers* are to be formed by inclosing five sheets of the coarse, in one of the finer quality; thus making in all twelve thicknesses of paper. These may be secured by a few stitches of small thread, which will render them less liable to be blown about by the wind, when exposed in the sunshine. It is more neat and convenient, to have the dryers precisely of the same dimensions as the press, though it is not essential. Four large newspapers folded within each other so as to present sixteen thicknesses of paper, make a very good dryer.

No trouble is experienced in transferring specimens from the port-folio to the press. Every thing was placed as it should be, in the field, while the plant was yet vigorous with life; and now the leaves and flowers, and even the minutest parts are disposed to keep their places. In a few minutes the port-folio may be emptied of its contents, which required days perhaps to collect. Place a dryer on a table, and upon it lay plants in such a manner that they do not overlay each other; cover them with a dryer and again lay on plants, and so on*. It is generally advisable to subject this bundle to fifteen or twenty pounds pressure, for a day or so, beneath a board, before it is admitted into the press. If the plants are not very juicy, as they generally are not towards autumn, or after protracted dry weather, this preliminary process may be dispensed with.

Although subjected to slight pressure only, in the course of twelve or twenty hours, the paper will become quite damp. The plants are now to be laid between dry papers, and placed in press; where, for a day or two, they may be subjected to a pressure varying from fifty to three hundred pounds. The next set of papers to which they are transferred, should be, if possible, absolutely dry. The pressure may now be unlimited; and generally the plants will be preserved the more beautiful, as the pressure is more considerable. The majority of plants

*That delicate specimens may be more easily removed from one set of dryers to another, they should be kept in single sheets of thin paper, as recommended by Dr. Short.

when thus treated, will become sufficiently dry in three or four days from the commencement of the process. Grasses, mosses, and ferns generally require much less time for drying than flowering plants; a single day being often sufficient to rid them entirely of moisture.

In regard to grasses, it is generally better that they should not be subjected to severe pressure; for it is desirable that the glumes should not be deformed, and that the leaves and culms, whether keeled or channeled, terete or triangular, should still retain their characteristic appearances. We should generally be satisfied then, with pressing grasses and their allies under a weight of ten or fifteen pounds.

When dryers have become damp from use, we are recommended to expose them in a favorable situation to sunshine. Some profess to be satisfied with spreading them upon the dry ground; but the flat roof of a shed, or a temporary floor of boards spread on the ground is decidedly preferable. If the dryers are not spread along in an imbricate manner, that is, if they are not permitted to overlap each other a little, it will be necessary to lay narrow strips of board upon them, to prevent the winds from blowing them away. They should be put out between the hours of ten in the morning and four in the afternoon; and if the weather be dry and the sky unclouded, two hours exposure will be sufficient. The most expeditious and effectual mode of drying papers, is by placing them around a fire. Two or three cords are to be stretched a few feet from a brisk fire, and the paper hung upon them. In eight or ten minutes they will require turning, and in fifteen or twenty minutes they will become more thoroughly dry than mere exposure to sunshine could ever render them. This plan has the advantage of being practicable in rainy weather, and I am satisfied that plants would be more beautifully preserved if it were always convenient to put it in requisition. In this manner I once rendered a parcel of common autumnal plants perfectly dry, in less than twenty-four hours; while the eye of the closest observer, could hardly discover that they had suffered the least appreciable change in color.

There are certain succulent, thick leaved vegetables, growing commonly in very dry situations, which for a considerable time resist the drying process; as Goodyera pubescens, Sedum ternatum, Erythronium americanum. Writers advise us to plunge them in boiling water, which by destroying the tenacious vital principle, materially abridges the time required for their desiccation. Specimens subjected to this ordeal invariably lose their natural color: it may sometimes be necessary, but I generally permit the drying to go on slowly, without scalding. In this patient manner, I have succeeded perfectly in preserving the characteristic colors of the species mentioned above.

When plants are sufficiently dry, they should be removed from the press, without any regard to the length of time they have been in. Although an opposite opinion has been expressed, I cannot conceive that there is any thing to be apprehended from rendering them too dry: on the other hand, I would recommend that they be perfectly dried in the first place, and ever after retained in that condition. Plants when moist, communicate a sensation of coldness to the fingers. A little experience will enable us to judge very correctly of this matter. When dried specimens are removed from the press, they may be put in sheets of wrapping paper, four or five specimens in a sheet, and it is of no importance if they overlay each other, if while in that condition, they be not subjected to pressure. They may then be placed in a trunk or drawer, and stored in an upper room, so that they may not contract dampness.

In changing his plants from one set of papers to another, as is necessary in the drying process, the student must not forget to accompany them with their appropriate labels. It may be well to attach the labels to one of the specimens in some rude manner, as by running the stem through a slit or two in the paper. It is easy to associate names with visible objects; and if the beginner will take the trouble to read the labels, when he has occasion to change the plants, he will be quite sure to connect them in his memory, almost without an effort.

The next subject to be considered, is the arrangement of specimens in some systematic manner, so that any particular species may be conveniently referred to.

### Directions for Arranging Dried Specimens.

Dr. Short recommends that the specimens be contained in sheets of paper, and not attached to them in any manner whatever; alleging as reasons, that they can be much more conveniently examined; that a better specimen may at any time be substituted for a more indifferent one; and that they can be more effectually protected from the injury of insects; not to take into account the time and trouble necessary to fix them, as recommended by Sir J. E. Smith, and others. I heartily concur with Dr. Short, in his general opinions relative to these matters; though I am rather inclined to disagree with him in some unimportant particulars: as for instance, in the quality of the paper to be used, the manner of attaching the labels, and the construction of cases to receive the papers and plants.

Those who wish to put up an herbarium in a neat and convenient manner, but of rather diminutive specimens, will find the common fools-cap paper exceedingly well adapted for their purpose. Well preserved specimens show to an admirable advantage, on fine white paper. The most *eligible* size of paper, perhaps, measures when doubled, ten inches by fourteen. It should be sized so that it may be written on,—very smooth and white, and moderately thick. If the plant be comparatively small, two, three, or more fellow species of a genus, can occupy the same paper. On the other hand, if necessary, the same individual species may occupy two or three papers. For my own part, I prefer keeping these papers with their contents, in the *cases* I am about to describe.

Sheets of paste-board are cut to the proper dimensions, and attached by an end and a side to strips of wood, as shown in the figure. They are best secured by small tacks; after which the wood and the nail heads may be concealed by pasting over thin leather, morocco, colored and polished paper, or starched cambric. These cases are intended to stand in a cabinet on the end A F, unless they are received into larger cases of similar construction, when their position would be reversed. They should be simply numbered in some manner upon the back. The smooth, ash colored press-board, such as is used by cloth dressers, is a very good material to construct them of; and if it be desired, after the boards are cut of the proper size, but before they are put together, one side may be covered with marbled, the other lined with fair white paper, which merely adds to their appearance without contributing to their utility. The width of the wooden strips in the direction A B, which determines the capacity of the case, should not much exceed half an inch. The student may be induced to remove the boards farther from each other, thereby enlarging the capacity; but he will invariably find the cases the less convenient for it. Plants should never be crowded into a case so as to distend it much; twenty or thirty papers in each, dependent upon the thickness of the specimens, will be sufficient. The corner D may be secured, by slipping over it a contrivance two or three inches square, equalling the case in the thickness, and of a construction very similar.

Larger boxes constructed on the same principle, of thick binder's boards, with the backs gilt and lettered, so as to resemble large folio volumes, will be found very convenient for receiving the smaller ones. If this method be adopted, the edges of the plant-bearing papers when in place, will not be visible. Each volume should contain five or six cases. The most eligible mode perhaps, is to dispense with the larger cases, keeping the smaller in a tight cabinet, the shelves of which are intersected at intervals of four or five inches, by vertical partitions.

Some diversity of sentiment has prevailed, in relation, to the system according to which plants should be arranged. There are some respectable botanists, who put up their collections in the alphabetical order of the genera.—This method certainly possesses the advantage of ready reference, in a pre-eminent degree; an advantage which will not fail of being appreciated, by the student who is not very familiar with the natural or artificial method of classification. This plan, however, necessarily associates the most dissimilar genera, as the graceful Monarda with the obscure Monila; and is consequently unfavorable to the acquirement of a true knowledge of nature. Though the Linnæan system does not offer the same advantage, it is liable to the same objection.—Take for instance, Monarda and Lycopus, which though inseparable from the mints, are yet made to occupy a place ten or twelve classes removed from them. I entirely concur in the opinion, that the Linnæan classification cannot be dispensed with so long as there are beginners in botany; and that it is the most eligible system for the arrangment of a *synopsis plantarum*, designed as a pocket companion, even for the accomplished botanist: yet I cannot discover the propriety of associating plants with the names of arbitrary classes and orders that really mean nothing. The Linnæan system is a contrivance for the sole purpose of conducting us easily to the name of a plant; and that once found, we have no farther use for it.

The professed object of the *natural method*, is to follow nature; to recognise those great orders or tribes making up the vegetable creation, which are distinguished by differences of general organization. Genera are grouped together, and orders defined, by characters drawn from the structure and functions of every organ and part, and a due estimate of their relative importance. Even the minute cellules and microscopic vessels that constitute the vegetable tissue, are not overlooked; and the general appearances, habits and qualities of plants are also taken into the account.

I cannot concur with Dr. Short in advising beginners to marshal their plants according to the system of Linnæus; on the other hand, however limited or extensive one's collection may be, I would recommend him to arrange it according to the natural system, as improved and adopted by De Candolle and Lindley; an exposition of which, sufficient for the purpose perhaps, will be found in Eaton's Manual, (6th ed.) though I think Lindley's order of arrangement deserves the preference. In reviewing his herbarium, the student will then acquire correct notions of philosophical botany: he will learn to appreciate those indications of relationship, which will often enable him upon first sight, to assign a plant he has never before seen its true place in the system, and to form a correct idea in regard to its hidden qualities.

The label must be written on the outside of the sheet, and supposing it to lie on an inclined desk, and to open towards the left, must be placed near the upper and right hand corner. If this be complied with, the labels can be referred to and read, without disturbing the plants, or removing the paper when in the case. Place first, and nearest the corner, the number of the natural order; then the name of the order; next the number and name of the sub-order, or section, if such there be; below this, the genus and species, referring to authority if necessary; after which the synonyms would be in order, but they may generally be omitted, if we select the most approved systematic name. Next the common or vulgar names, and lastly the locality and date; to which may be appended any brief observations thought necessary.

The following diagram will render the matter plain:

221
LABIATÆ.
§5. Nepeteæ.
*Galeopsis tetrahit.*
Flowering nettle.
RIVER BANK, OGDENSBURG, N. Y.
*July 5th*, 1831.

When several plants occur under the same order or section, the name may be omitted but the number retained. It is of less consequence to particularize the year, than to note the month in which the plant was collected. If several species of a genus occupy the same paper, the labels must be numbered, and small bits of numbered papers attached to the corresponding specimens. For this purpose, a little paste, mucilage of gum Arabic, or surgeon's adhesive plaster may be used.

Having the plants all duly labeled and arranged in the numbered cases, we have next to make an index, simply by taking an inventory of the orders contained in each case. This may be pasted for convenience upon a piece of press-board, or upon the inner side of one of the cabinet doors. It might also sometimes be convenient to have at hand an alphabetical list of orders, each with its number annexed. Until the student become familiar with the arrangement of his herbarium, when he wishes to find a particular plant, he will refer to the genus in Eaton's Manual, or Torrey's Catalogue, where he will observe the number of the order to which the plant belongs.

If we would preserve unchanged, the beauty and freshness of a collection for a series of years, we must take measures to preclude moisture, and prevent the depredation of insects.

My own experience does not exactly accord with that of Sir J. E. Smith, in relation to the danger to be apprehended from insects; which may perhaps, in part be owing to climate, or what is more probable, to the rapid and efficient manner in which most of my specimens have been dried. I have never discovered that any of them are infested by the *Ptinus fur.* In those papers not scented with camphor or volatile oil, I have frequently noticed an *apterous* insect much more minute, which is barely visible to the naked eye at the distance of eighteen or twenty inches. The immediate mischief occasioned by them is by no means very serious; doubtless in a few years it could be more easily appreciated.

A very neat, successful, and convenient method of defending the herbarium from the encroachment of insects, is to remove each specimen temporarily, and sprinkle the fine powder of camphor upon that part of the paper which it covered. The process may be repeated as often as there is necessity for it.

One cannot observe too much nicety, in keeping a collection dry. The cabinet or trunk containing it, should be placed in an upper room, and must never be opened in damp weather, unless the air of the apartment be first rendered dry by a fire. If the climate be habitually damp, it would be advisable to line the cabinet entirely with tin plate, and list the doors with strips of gum elastic. It is an excellent practice to keep unslaked lime contained in a jar or paper, in the trunk or cabinet with the plants. It will not require renewing often if the cabinet be moderately tight. The lime keeps the air perfectly dry, in which condition it can be more completely impregnated with the vapor of camphor and volatile oils, and thereby effectually defended from insects.

The directions of Dr. Short, relative to *exchanges*, (*Instructions for making Herbaria. Lexington*, 1833,) are so entirely those which I would wish to give, that I shall offer no apology for adopting them.

"It should be the constant aim of every botanist, not only to increase his own knowledge, by every possible opportunity, but to add something to the general stock; and this is most readily and effectually done, by a free and liberal interchange of specimens with other botanists. * * * Thus will your collection be constantly enriched, by new acquisitions, and in proportion to the number of species in each genus which you get together, will you find it more and more easy to identify with positive certainty, any plant you meet with, by comparing it with those you have. Of such as you are unable to ascertain the name by reference to the proper books, it will be only necessary to affix corresponding numbers to the specimens which you reserve, and those you send to your correspondent, who, if a competent botanist, will thus be enabled to furnish you the names. All specimens intended for the purposes of exchange, should be well secured in a light and tight box, of the exact size with the paper in which the plants are included; the top of which should make considerable pressure upon them, when fastened down, so as to prevent all displacement of specimens or labels, by transportation. * * * In this way, you will contribute essentially towards the formation of that desideratum in our Botany—*an accurate and complete Flora of the American Union.*"

## Figures and Tables

**Figure f1:**
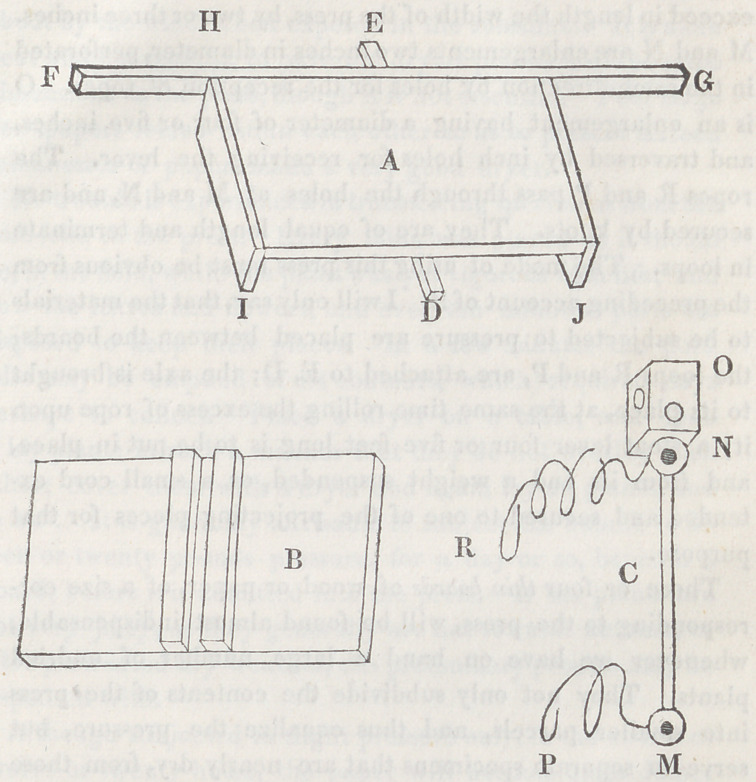


**Figure f2:**